# Noninvasive Tracking of Donor Cell Homing by Near-Infrared Fluorescence Imaging Shortly after Bone Marrow Transplantation

**DOI:** 10.1371/journal.pone.0011114

**Published:** 2010-06-14

**Authors:** Takashi Ushiki, Shinae Kizaka-Kondoh, Eishi Ashihara, Shotaro Tanaka, Masayoshi Masuko, Hideyo Hirai, Shinya Kimura, Yoshifusa Aizawa, Taira Maekawa, Masahiro Hiraoka

**Affiliations:** 1 Department of Radiation Oncology and Image-Applied Therapy, Kyoto University Graduate School of Medicine, Kyoto, Japan; 2 Department of Hematology, Niigata University Graduate School of Medical and Dental Sciences, Niigata, Japan; 3 Department of Transfusion Medicine and Cell Therapy, Kyoto University Hospital, Kyoto, Japan; 4 Department of Internal Medicine, Faculty of Medicine, Saga University, Saga, Japan; 5 Department of Cardiology, Niigata University Graduate School of Medical and Dental Sciences, Niigata, Japan; University of Birmingham, United Kingdom

## Abstract

**Background:**

Many diseases associated with bone marrow transplantation (BMT) are caused by transplanted hematopoietic cells, and the onset of these diseases occurs after homing of donor cells in the initial phase after BMT. Noninvasive observation of donor cell homing shortly after transplantation is potentially valuable for improving therapeutic outcomes of BMT by diagnosing the early stages of these diseases.

**Methodology/Principal Findings:**

Freshly harvested near-infrared fluorescence-labeled cells were noninvasively observed for 24 h after BMT using a photon counting device to track their homing process. In a congenic BMT model, the homing of Alexa Fluor 750-labeled donor cells in the tibia was detected less than 1 h after BMT. In addition, subsequent cell distribution in an intraBM BMT model was successfully monitored for the first time using this method. In the allogeneic BMT model, T-cell depletion decreased the near-infrared fluorescence (NIRF) signals of the reticuloendothelial system.

**Conclusions/Significance:**

This approach in several murine BMT models revealed that the transplanted cells homed within 24 h after transplantation. NIRF labeling is useful for tracking transplanted cells in the initial phase after BMT, and this approach can contribute to in vivo studies aimed at improving the therapeutic outcomes of BMT.

## Introduction

Bone marrow transplantation (BMT) is an important procedure for curing hematological malignancies, although engraftment failure [1] and graft-versus-host disease (GVHD) [2] remain serious complications following allogeneic BMT. To examine these complications, it is important to monitor the transplanted donor cells in the initial phase after BMT because donor cell homing is a rapid process. Homing is defined as the anchoring of hematopoietic cells in their niche before cell proliferation [3]. Results of a colony-forming assay revealed that donor cell homing occurs within minutes or a few hours, rather than days, after BMT [4]. BMT-associated complications are suspected to begin just after homing of the donor cells; for example, in studies of engraftment failure, homing of hematopoietic stem cells (HSCs) is crucial and is the first step in hematopoietic reconstitution [5]. In acute GVHD (aGVHD), T-cell activation by host antigen-presenting cells [6], [7] begins after homing in the reticuloendothelial system, and this process starts within a few days after BMT in murine GVHD models [8].

Recent molecular imaging techniques have facilitated significant advances in noninvasive optical imaging [9], which enable tracking of transplanted hematopoietic cells with greater accuracy in vivo. Bioluminescence imaging can precisely analyze the processes during BMT [10]. However, until now, bioluminescence imaging has provided sparse spatiotemporal information for donor cell homing in the initial phase after BMT.

In this study, we directly labeled donor BM cells using a near-infrared fluorescence (NIRF) dye with high tissue permeability and monitored the homing of transplanted cells shortly after BMT using a noninvasive whole-body imaging device IVIS-Spectrum. The overall results provide information regarding the homing of donor cells after BMT and intraBM-BMT (IBM-BMT), and the onset of GVHD. Noninvasive tracking of transplanted donor cells in the initial phase after BMT enables acquisition of spatiotemporal information regarding HSC homing, which can help identify factors supporting HSC homing and mechanisms of hematopoietic reconstitution.

## Materials and Methods

### Ethics statement

All animal experiments in this study were performed with the approval of the Animal Experiment Committees of Kyoto University, Graduate School of Medicine. The approval number of the experiment is Med Kyo 09247. Approved experiments included use of transgenic mice and primary cells, cell transplantation, in vivo and ex vivo optical imaging, and UV irradiation.

### Mice

Balb/c nu/nu (H-2^d^, Thy 1.2), Balb/c (H-2^d^, Thy 1.2), C57BL/6 (H-2^b^, Thy 1.2), and FVB/N (H-2^q^, Thy 1.1) mice were purchased from Japan SLC Inc. (Hamamatsu, Japan). Enhanced green fluorescent protein (EGFP) transgenic mice were generated by injecting the CAAG-EGFP expression vector into a one-cell embryo of an ICR closed colony (ICR/EGFP) [11], followed by breeding in an SPF animal facility. All mice were 7–9 weeks of age and were fasted according to our original fasting protocol from the day before BMT until 24 h after BMT (Supplemental Fig. S1) for suppression of autofluorescence from food particles in the gastrointestinal (GI) tract.

### Cell preparation for transplantation and in vitro assays

BM cells were prepared from the medullary cavities of the humerus, femur, and tibia. Splenocytes were prepared by homogenization of the spleen. BM mononuclear cells (BM-MNCs) and spleen mononuclear cells (Sp-MNCs) were obtained by density gradient centrifugation using Lympholyte-M solution (Cedarlane Labs, Hornby, ON, Canada). T-cell-depleted (TCD) BM-MNCs were obtained by negative selection using Thy1.2^+^ microbeads (Miltenyi Biotech, Auburn, CA, USA).

### NIRF and 5-(6)-carboxyfluorescein diacetate succinimidyl ester (CFSE) labeling

Cy5.5 monofunctional dye (peak excitation: 675 nm, peak emission: 694 nm; GE Healthcare UK Ltd, Buckinghamshire, UK) and Alexa Fluor 750 carboxylic acid, succinimidyl ester (AF750) (peak excitation: 749 nm, peak emission: 775 nm; Invitrogen, Eugene, OR, USA) were dissolved in N, N-dimethylformamide. To prepare Cy5.5- or AF750-labeled cells, BM-MNCs and Sp-MNCs were incubated with Cy5.5 (0.4 mg/mL) or AF750 (0.1 mg/mL) for 15 min at 37°C under 5% CO_2_. The cells were washed twice with phosphate-buffered saline (PBS) and then once with 10 mM Tris in PBS to disturb the active groups. For CFSE (Invitrogen) labeling, the cells were incubated in 5 µM CFSE in PBS for 10 min at 37°C, followed by washing with excess ice-cold PBS on ice for 5 min to quench staining. The cells were then washed 3 times with PBS. To prepare CFSE and NIRF double-positive cells, CFSE staining was performed before NIRF labeling.

### Cell proliferation assays

NIRF-labeled Sp-MNCs (1×10^5^) were seeded in a 96-well plate with 100 µL of phenol red-free RPMI 1640 (Wako Pure Chemical Industries Ltd., Osaka, Japan) supplemented with 1 mM sodium pyruvate, 10 mM HEPES, 100 units/mL penicillin, 100 µg/mL streptomycin, 50 µM mercaptoethanol, and 10% FCS. To stimulate splenocyte proliferation, recombinant mouse IL-2 (mIL-2) (20 ng/mL; R&D Systems, Minneapolis, MN, USA) and anti-CD3/CD28 antibody-coated beads (Dynabeads mouse CD3/CD28 T-cell expander; Invitrogen Dynal AS, Oslo, Norway) were added to the medium. The CD3/CD28 T-cell expander was used at a 1∶1 bead-to-cell ratio according to the manufacturer's instructions. The viable cell number was analyzed as previously described [12] with slight modifications. The absorbance (450 nm) was measured 3 h after adding the cell-counting reagent.

### Analysis of clonogenic myeloid and erythroid progenitors

BM cells (2×10^4^) were harvested from 6- to 8-week-old Balb/c nu/nu mice and cultured in Methocult GF M3434 (Stem Cell Technologies, Vancouver, BC, Canada) according to the manufacturer's instructions after NIRF labeling. Granulocyte-macrophage colony-forming units (GM-CFUs); erythroid burst-forming units (BFU-Es); and granulocyte, erythrocyte, macrophage, and megakaryocyte colony-forming units (GEMM-CFUs) were scored after 7 days in culture.

### Fluorescence-activated cell sorter (FACS) analyses

FACS data were obtained using a Canto II flow cytometer (Becton Dickinson, Mountain View, CA, USA) and analyzed using FACSDiva (Becton Dickinson) or FlowJo software (TreeStar, Ashland, OR, USA). A red helium-neon (633 nm) laser was used for excitation of Cy5.5 (analyzed using an APC filter) and AF750 (analyzed using an APC-Cy7 filter). NIRF-labeled ICR/EGFP BM-MNCs were used for analysis of chimerism of transplanted cells. Antibodies against FITC-conjugated Thy1.1 (CD90.1) and PE-conjugated Thy1.2 (CD90.2) were purchased from BD Pharmingen, San Diego, CA, USA. AF750-labeled Balb/c Thy1.2^+^ cells (1×10^6^) were mixed with unlabeled FVB/N Thy1.1^+^ cells (1×10^6^) in a 1∶1 ratio and incubated together in PBS at 37°C for 3 h, and the AF750 intensity of Thy1.1^+^ and Thy1.2^+^ cells was determined. We estimated the percentages of EGFP^+^ cells in the tibiae and peripheral blood of the recipients at 24, 48, and 72 h after BMT. Erythrocytes were removed using ACK lysis buffer (Invitrogen). DNA content was analyzed as previously described [13].

### Pathological examinations

For ex vivo analyses of the chimerism of transplanted EGFP^+^ cells in the tibiae, the recipient mice were sacrificed at 24, 48, or 72 h after BMT. BM cells were flushed from the tibiae using PBS and dyed with 100 µg/mL DAPI (Sigma-Aldrich, St. Louis, MO, USA). These cells were then observed with an inverted fluorescent microscope. To detect EGFP^+^ cells in the organs after BMT, the Balb/c nu/nu mice receiving ICR/EGFP BM-MNCs were sacrificed at 24, 48, or 72 h after BMT. Organs from the mice were then removed, fixed in 10% formalin, and embedded in paraffin. Furthermore, 3-µm-thick sections were deparaffinized and incubated with blocking solution (1% Block Ace; Dainippon Pharmaceutical Co. Ltd., Osaka, Japan) for 15 min at room temperature. Sections were incubated with polyclonal rabbit anti-EGFP antibody (1∶1000 dilution in PBS; Abcam, Cambridge, MA, USA) at 4°C overnight and then with FITC-conjugated swine anti-rabbit immunoglobulin (1∶30 dilution in PBS; Dako, Glostrup, Denmark) for 1 h at room temperature. They were then washed and mounted in VECTASHIELD with DAPI (Vector Laboratories, Burlingame, CA, USA). All photos were taken using a BZ-9000 microscope (KEYENCE, Osaka, Japan).

### Transplantation of donor cells

Balb/c nu/nu mice (7–9 weeks old) or Balb/c mice (7–9 weeks old) were lethally irradiated (8 Gy) in a single fraction by a ^137^Cs γ-ray using a Gammacell 40 Exactor (MDS Nordion International Inc., Ontario, Canada). BM-MNCs and Sp-MNCs were injected into the recipient mice through the tail vein at 6–8 h after irradiation. For ex vivo analyses of the chimerism of transplanted EGFP^+^ cells in the tibiae or peripheral blood, 1×10^7^ Cy5.5- or AF750-labeled ICR/EGFP BM-MNCs were transplanted into the Balb/c nu/nu mice. The recipient mice were sacrificed at 24, 48, or 72 h. For in vivo imaging with congenic BMT, 1×10^7^ AF750-labeled Balb/c nu/nu BM-MNCs were injected into the Balb/c nu/nu mice. For allogeneic BMT, 1×10^7^ AF750-labeled C57BL/6 BM-MNCs, 1×10^7^ AF750-labeled TCD C57BL/6 BM-MNCs, or 5×10^6^ AF750-labeled C57BL/6 TCD BM-MNCs plus 5×10^6^ AF750-labeled C57BL/6 Sp-MNCs were injected into 7- to 9-week-old Balb/c nu/nu mice.

### IBM-BMT model

IBM-BMT was performed as described previously [14]. In brief, 7- to 9-week-old Balb/c nu/nu mice were lethally irradiated (8 Gy) in a single fraction by a ^137^Cs γ-ray 24 h before BMT. For IBM-BMT, the mice were then anesthetized with isoflurane, and the left tibia was gently drilled using a 26-G microsyringe (50 µL; Hamilton, Reno, NV, USA) through the patellar tendon. The AF750-labeled BM-MNCs (1×10^7^ in 30 µL PBS) were directly injected into the BM cavity thereafter.

### In vivo and ex vivo imaging of transplanted mice

In vivo imaging was performed using an IVIS Spectrum system (Xenogen, Alameda, CA, USA) at 5, 15, or 30 min and 1, 3, 6, 12, 18, or 24 h after BMT. During imaging, the mice were kept on the imaging stage under anesthesia with 2.5% isoflurane gas in oxygen flow (2 L/min). Transplanted Cy5.5- or AF750-labeled cells were detected using emission and excitation filters (excitation/emission: 640/700 for Cy5.5-labeled cells, 710/780 for AF750-labeled cells). The conditions were as follows: exposure time  = 5 s, lamp level  =  high, binning  =  medium, field of view  = 12.9×12.9 cm, and f/stop  = 1. Ex vivo imaging was performed using the IVIS Spectrum system 24 h after BMT, under the same conditions as for in vivo imaging. For free NIRF injection, free Cy5.5 (625 ng) or AF750 (40 ng) in 100 µL Tris in PBS (10 mM), with the same fluorescence intensity as 1×10^7^ NIRF BM-MNCs, was injected through the tail vein.

### Statistical analysis

Student's *t* tests were used to determine the statistical significance. *P*<0.05 was considered significant.

## Results

### Direct NIRF labeling technique shows low cytotoxicity

**Figure 1:**
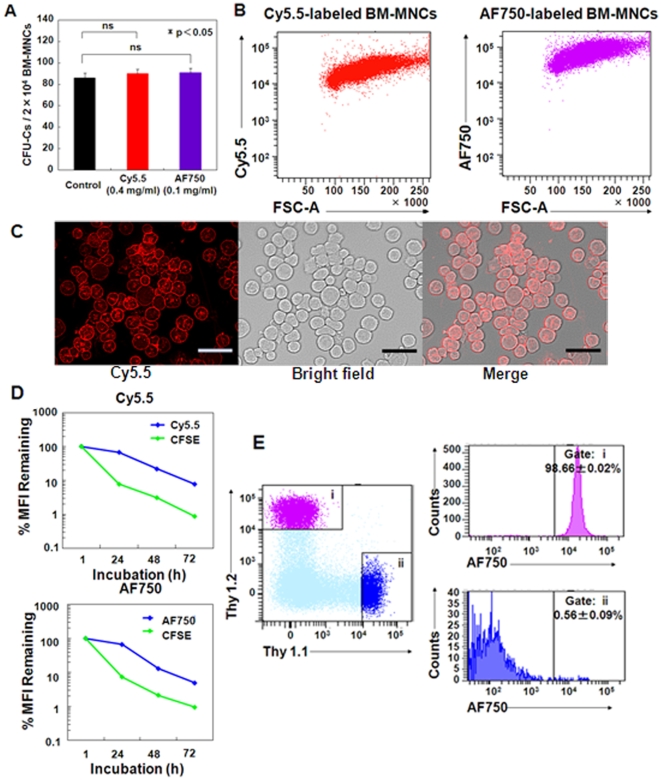
Evaluation of cytotoxicity and in vitro fluorescence intensity of NIRF labeling. (A) GM-CFUs, BFU-Es, and CFU-GEMMs were counted (n = 5); mean CFUs per 2×10^4^ BM-MNCs are shown. (B) FACS analysis of Balb/c nu/nu BM-MNCs labeled with Cy5.5 (0.4 mg/mL) and AF750 (0.1 mg/mL). (C) Representative fluorescence image of Cy5.5-labeled Balb/c nu/nu BM-MNCs. Bar  = 100 µm. (D) In vitro retention of NIRF and CFSE in Balb/c Sp-MNCs for the indicated times after labeling (n = 8). Sp-MNCs (1×10^5^) were seeded in a 96-well plate with phenol red-free RPMI 1640 medium containing CD3/28 beads and mIL2. Values are %MFI ± SEM. Error bars are less than 1.1%. (E) AF750-labeled Balb/c Thy1.2^+^ Sp-MNCs (1×10^6^) (i) were mixed in a 1∶1 ratio with unlabeled FVB/N Thy1.1^+^ Sp-MNCs (1×10^6^) (ii) at 37°C for 3 h, and the AF750 intensity of Thy1.1^+^ and Thy1.2^+^ cells was analyzed by FACS.

Since most diseases associated with BMT are caused by transplanted hematopoietic cells, it is very important to label all the donor cells. Freshly harvested BM-MNCs from Balb/c nu/nu mice were labeled with 0.4 mg/mL Cy5.5 and 0.1 mg/mL AF750, which did not show obvious cytotoxicity or growth inhibition (Supplemental Figs. S2 and S3). Furthermore, the NIRF dyes did not affect the colony-forming efficiency at these concentrations (Fig. 1A). BM-MNCs were efficiently labeled regardless of cell size and diversity (Fig. 1B). Using the NIRF dyes, homogeneous labeling on the cell surfaces was achieved despite using a variety of cell populations (Fig. 1C).

### Fluorescence on the cell surface is retained and does not influence surface antigen recognition

Balb/c Sp-MNCs were double-stained with CFSE and NIRF dyes, and their mean fluorescence intensity (MFI) was measured every 24 h until 72 h using FACS (Fig. 1D). To activate and expand Sp-MNCs, we cultured them with CD3/28 beads and mIL-2. MFI of CFSE decreased by 1 log during the first 24 h, while MFI of NIRF hardly changed for 24 h; it was halved following cell division after 48 h. Because Sp-MNCs were labeled using the same method, the difference in MFI between CFSE and NIRF can be attributed to the faster fluorescence decay of CFSE, as its excitation wavelength is in the visible range, rather than a difference in dye retention time on the cell surface. These results indicate that NIRF retains more stable MFI than CFSE.

During in vivo fluorescence imaging, recipient mice received approximately 20 excitations over a 24-h period. We confirmed that repeated excitations did not influence cell viability (Supplemental Fig. S4) or the NIRF MFI on the cell surface (Supplemental Fig. S5). It was also considered that precise evaluation of the obtained fluorescence images would be influenced by transfer of the NIRF dye from the donor cells to neighboring recipient cells after BMT and that the NIRF dyes on the cell surface would hinder recognition of cell surface markers. To examine these possibilities, we co-cultured Balb/c-derived Thy1.2^+^ Sp-MNCs (NIRF labeled) and FVB/N-derived Thy1.1^+^ cells for 3 h and examined their NIRF-labeling status and recognition by corresponding antibodies using FACS. The cells labeled with NIRF were recognized as Thy1.2^+^. Co-cultured Thy1.1^+^ cells showed no increase in NIRF (Fig. 1E). These results indicate that NIRF dyes were universally retained on the cell surface and did not influence the recognition of cell surface antigens, suggesting that in vivo fluorescence imaging with NIRF-labeled cells would contribute to acquisition of spatiotemporal information regarding donor cells shortly after BMT.

### In vivo tracking of Cy5.5-labeled hematopoietic cells during the first 24 h after BMT

**Figure 2:**
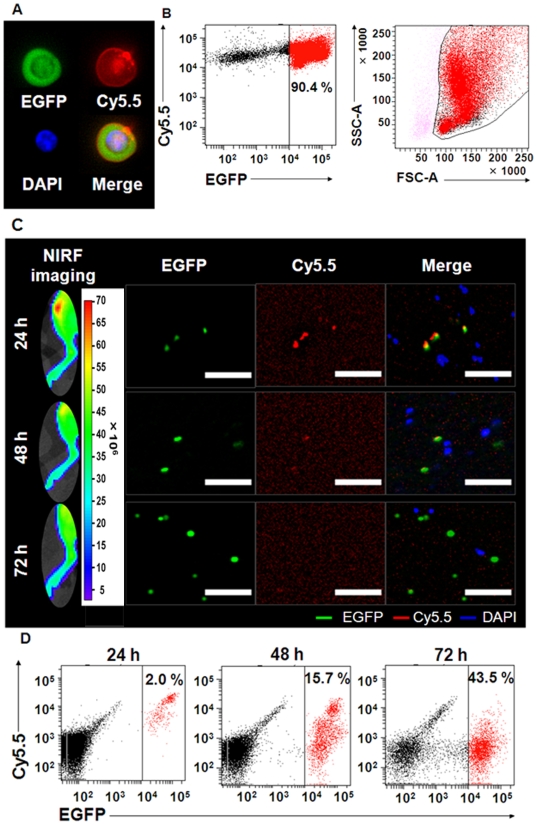
EGFP and Cy5.5 double-positive cells were detected in BM at 24 h after BMT. (A) Representative fluorescence image of Cy5.5-labeled ICR/EGFP BM-MNCs. Green, EGFP; blue, DAPI; red, Cy5.5. (B) FACS analysis of Cy5.5-labeled ICR/EGFP BM-MNCs at 1 h after labeling. The EGFP^+^ cells included all leukocyte fractions. (C) Analysis of Cy5.5 and EGFP double-positive cells in the tibiae at the indicated times. Recipient Balb/c nu/nu mice (H-2^d^) were transplanted with 1×10^7^ Cy5.5-labeled ICR/EGFP BM-MNCs (closed colony). In vivo fluorescence imaging of the right legs of mice transplanted with Cy5.5-labeled BM-MNCs obtained from ICR/EGFP mice is shown (left column). The cells from the tibiae were observed under an inverted fluorescent microscope (right column). Bar  = 40 µm. Green, EGFP; blue, DAPI; red, Cy5.5. (D) FACS analysis of chimerism of Cy5.5 and EGFP double-positive cells in the tibiae at the indicated times.

Cy5.5-labeled Balb/c nu/nu BM-MNCs were transplanted to the Balb/c nu/nu recipient mice. Since abdominal autofluorescence caused by food decreases the precision of in vivo imaging, all mice were fasted before in vivo imaging to suppress autofluorescence from food particles in the GI tract, (Supplemental Figs. S1 and S6). The Cy5.5 signal was observed from both sides of the tibiae and spines at 5 min after BMT through the tail vein (Supplemental Fig. S7). We further examined the status of transplanted BM-MNCs with Cy5.5 labeling (Fig. 2) and found that BM-MNCs from ICR/EGFP were homogeneously labeled with Cy5.5 on the cell surface (Fig. 2A). FACS analysis revealed that the EGFP^+^ cells included all leukocyte fractions (Fig. 2B).

To confirm whether the in vivo Cy5.5 signals from the tibia (Supplemental Fig. S7) were derived from Cy5.5-labeled transplanted BM-MNCs, we transplanted Cy5.5-labeled ICR/EGFP BM-MNCs to the Balb/c nu/nu mice. Cy5.5 signals were observed in the tibia 24 h after BMT; these signals decreased thereafter (Fig. 2C). BM cells in the tibia were flushed and then observed by fluorescence microscopy on the same schedule as for in vivo imaging. We confirmed that the Cy5.5 signals detected in the tibia 24 h after BMT were from the transplanted BM-MNCs and belonged to the Cy5.5^+^ and EGFP^+^ cell populations (Fig. 2C, upper row). The cell surface Cy5.5 signal decreased throughout the experiment, probably due to cell division (Fig. 2C), while the chimerism of the EGFP^+^ cells increased rapidly (Fig. 2D). The NIRF signals emanating from the transplanted donor cells decreased with inverse proportion to cell division.

### Evaluation of in vivo imaging of NIFR-labeled donor cells revealed that the NIRF tracking approach is highly sensitive

**Figure 3:**
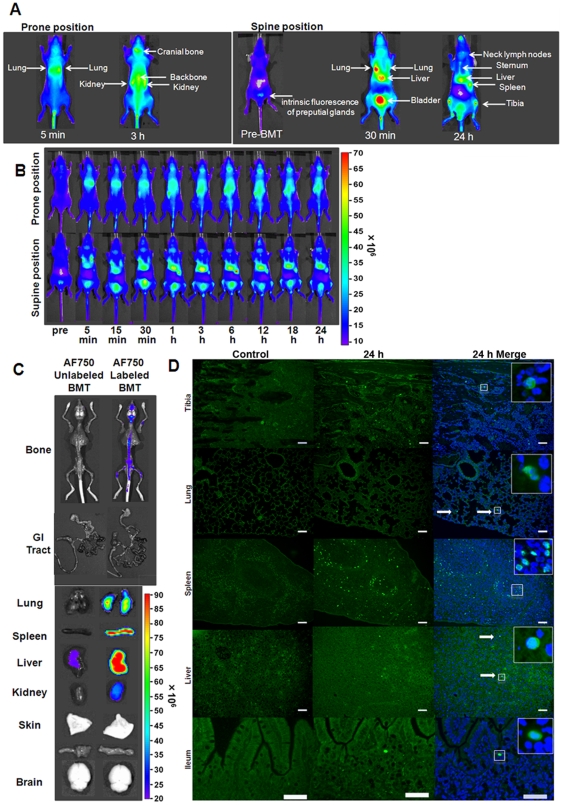
Fluorescent signals from AF750/EGFP double-positive cells were detected in vivo with extremely high sensitivity. Lethally irradiated Balb/c nu/nu (H-2^d^) mice were transplanted with 1×10^7^ AF750-labeled ICR/EGFP BM-MNCs. (A) Sources of NIRF signals in vivo. The congenic BMT model is shown as an example. (B) Representative in vivo images of the recipient mice at the indicated times after BMT. The prone (upper row) and supine (bottom row) positions are shown. (C) Ex vivo imaging of the recipient mice. Mice transplanted with AF750-labeled (left) or unlabeled (right) BM-MNCs were sacrificed at 24 h after BMT. The congenic AF750-unlabeled BMT model is shown as a control. (D) Fluorescence images of various organs from recipient mice. AF750-labeled ICR/EGFP BM-MNCs were transplanted, and the organs were examined 24 h after BMT. Untreated Balb/c nu/nu mice were used as controls. Green, EGFP; blue, DAPI. White arrows show EGFP/DAPI double-positive cells. Bar  = 100 µm.

Next, we transplanted AF750-labeled ICR/EGFP BM-MNCs into the Balb/c nu/nu mice to evaluate the sensitivity and accuracy of our approach for tracking the homing process of donor cells in vivo. Distribution of the donor cells was monitored using an in vivo imaging device for 24 h after BMT. Using AF750-labeled cells, transplanted cells were detected in many organs in vivo (Fig. 3A). The distribution of donor cells was spread over several organs including the lung, liver, spleen, and BM (Fig. 3B). Ex vivo imaging of the recipient mice at 24 h after BMT confirmed NIRF signal distribution in the tissues suggested by in vivo imaging (Fig. 3C). Significant NIRF signals were detected in BM and the reticuloendothelial system, but not in the GI tract (Fig. 3C). To confirm the presence of donor cells in the tissues detected by ex vivo fluorescent imaging, sections of the tissues were examined for presence of EGFP^+^ cells 24 h after transplantation (Fig. 3D). Although significant fluorescent signals were detected in the tissues by in vivo and ex vivo imaging, the EGFP^+^ cells were sparsely distributed in each tissue, indicating that in vivo NIRF imaging is highly sensitive.

### In vivo and ex vivo tracking of AF750-labeled cells in various congenic BMT models in an initial phase after transplantation

**Figure 4:**
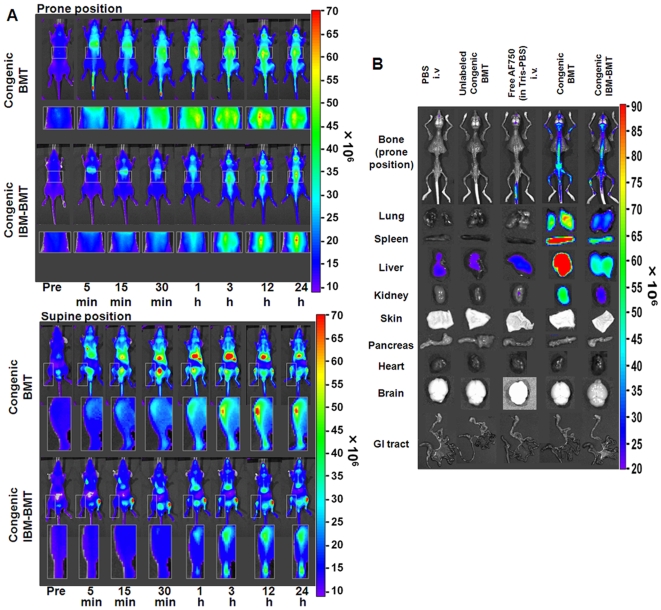
In vivo imaging of transplanted AF750-labeled donor cells in various congenic BMT models. (A) In vivo imaging of congenic BMT models until 24 h after BMT. In the congenic BMT model, the transplanted cells were detected in the tibiae at 5 min after congenic BMT (supine position). In the congenic IBM-BMT model, the migrating cells from the left tibia were detected 1 h after congenic IBM-BMT. The parts of the body with significantly increased signals (indicated by squares) were magnified and are shown below the corresponding whole-body images. (B) Ex vivo imaging of transplanted AF750-labeled donor cells in the BMT models at 24 h after BMT.

The above results strongly suggest that in vivo imaging of NIRF-labeled donor cells would provide a highly sensitive and accurate method to track the homing process of transplanted cells. First, congenic BMT was performed to evaluate the homing process of transplanted cells in the absence of rejection. Biodistributions of the transplanted BM-MNCs labeled with AF750 were then compared in various congenic BMT models at an early stage after transplantation. The AF750 images obtained after injection of NIRF-unlabeled BM-MNCs were identical to those of the background signals in PBS-injected mice (Supplemental Fig. S8). After intravenous injection of free AF750, the fluorescent signal disappeared through the urinary system within 1 h after BMT (Supplemental Fig. S8). In the congenic BMT model mice, the fluorescent signal was detectable in both tibiae 5 min after transplantation. It gradually increased by 3 h and remained unchanged for the rest of the observation period (Fig. 4A, supine position). We detected transplanted cell homing in vivo at 5 min after BMT, which is the earliest detection time reported thus far. In the reticuloendothelial system, the transplanted cells were first seen trapped in the lung 5 min after BMT and then in the liver and spleen 15 min after BMT. In the IBM-BMT model, the transplanted cells were seen trapped in the lung through the peripheral circulation 5 min after IBM-BMT (Fig. 4A, supine position). In this model, the donor cells migrated from the left tibia to the right one within 1 h after BMT (Fig. 4A, supine position), although many cells stayed in the left tibia for 24 h. Tissue distribution of the fluorescent signal was examined by ex vivo imaging 24 h after BMT in both BMT models. When the free AF750 dye was injected intravenously, the background level slightly increased in each organ, although the increase was negligible (Fig. 4B). In a comparison of congenic BMT with congenic IBM-BMT (Fig. 4B), most of the donor cells were found to remain in the injected left tibia in congenic IBM-BMT mice, while most of the donor cells migrated to all bones of the body in the congenic BMT mice. To our knowledge, this is the first report using in vivo imaging to reveal the distribution dynamics of transplanted cells after IBM-BMT, and the results suggest that the NIRF labeling approach would be applicable in various congenic BMT models.

### Transplanted donor cell division starts more than 24 h after BMT

**Figure 5:**
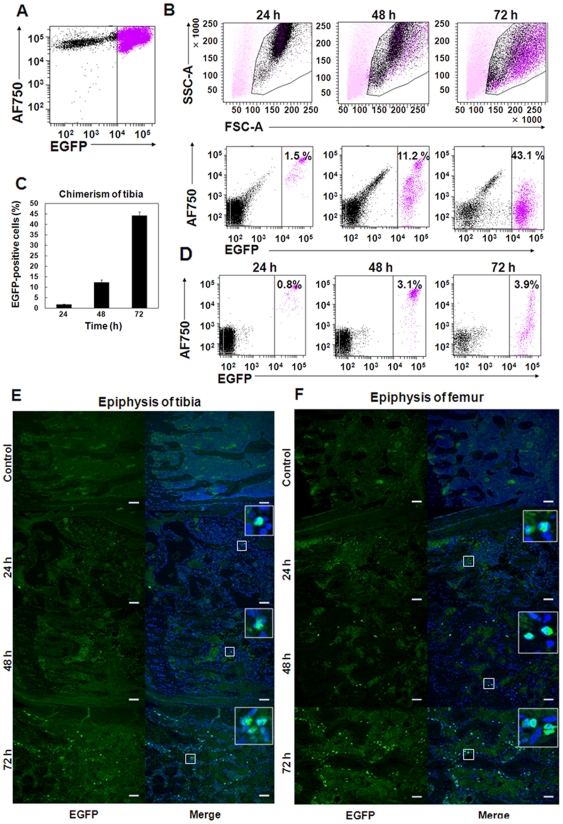
Homing donor cells start cell division at least 24 h after BMT. (A) FACS analysis of AF750-labeled donor ICR/EGFP BM-MNCs was performed immediately after labeling. (B) Chimerism of the transplanted donor AF750/EGFP double-positive cells in the tibiae. Lethally irradiated Balb/c nu/nu recipients (H-2^d^) were transplanted with 1×10^7^ AF750-labeled ICR/EGFP BM-MNCs. The cells in the tibiae were harvested, and EGFP^+^ cells were analyzed by FACS at the indicated times after BMT. (C) Mean% ± SEM of EGFP^+^ cells in the recipient tibiae at the indicated times after BMT (n = 4). (D) The chimerism of EGFP^+^ cells in the peripheral blood of the mice in (B) was analyzed by FACS. (E, F) Representative fluorescence images of the tibiae (E) and femora (F). The bones were removed from the mice at the indicated times after BMT. Slices (3-µm thick) of the tibial and femoral epiphyses were observed with an inverted fluorescent microscope. Donor EGFP^+^ cells rapidly expanded in the tibial and femoral epiphyses between 48 and 72 h after BMT. Untreated Balb/c nu/nu mice were used as controls. Green, EGFP; blue, DAPI. Bar  = 100 µm.

To determine the start time of transplanted donor cell division after homing, we harvested the cells from the tibiae and peripheral blood of the recipient mice at 24, 48, and 72 h after transplantation of AF750-labeled ICR/EGFP BM-MNCs and examined the fraction of EGFP^+^ cells present by FACS (Fig. 5A). The NIRF intensity of EGFP^+^ cells 24 h after BMT was similar to that of preBMT cells (Figs. 5A, B second row), indicating that the donor cells had not started cell division until then because NIRF intensity decreased with cell division (Fig. 1D, Supplemental Fig. S3). Donor cell division started thereafter because the chimerism of EGFP^+^ cells in the tibiae of the recipients increased by approximately 45% at 72 h after BMT (Figs. 5B, C). FACS analysis revealed that the donor cell population contained all leukocyte fractions at 72 h after BMT (Fig. 5B, top right panel) and that EGFP^+^ appeared in the peripheral blood at 72 h with similar fluorescence intensity as that in BM (Fig. 5D, Supplemental Fig. S9).

Immunohistochemical analysis confirmed that the number of transplanted EGFP^+^ cells did not increase until 24 h after BMT and then rapidly expanded in the epiphyseal area of the tibia and femur between 48 and 72 h after BMT (Figs. 5E, F). These results indicate that transplanted donor cell division starts more than 24 h after BMT and that hematopoietic reconstitution in Balb/c nu/nu recipient mice begins approximately 72 h after BMT.

### In vivo and ex vivo tracking of AF750-labeled cells in various allogeneic BMT models

**Figure 6:**
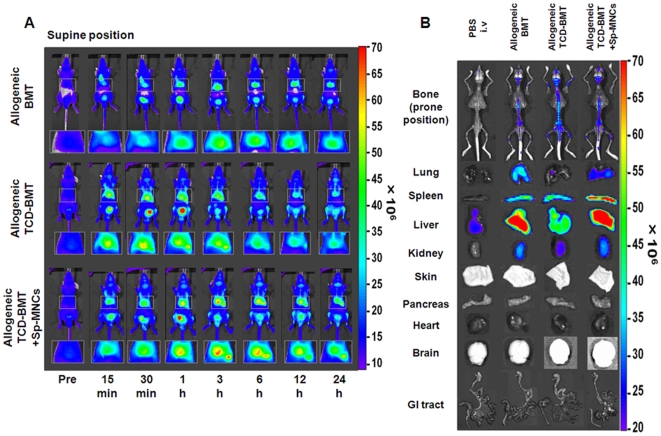
In vivo imaging of transplanted AF750-labeled donor cells in various allogeneic BMT models. (A) In vivo imaging of allogeneic BMT models until 24 h after BMT. Conditioned Balb/c nu/nu recipients (H-2^d^) received 1×10^7^ AF750-labeled C57BL/6 BM-MNCs (H-2^b^) (upper row), 1×10^7^ TCD C57BL/6 BM-MNCs (middle row), or 5×10^6^ C57BL/6 TCD BM-MNCs plus 5×10^6^ C57BL/6 Sp-MNCs (bottom row). Areas indicated by squares are magnified and shown below each image. (B) Ex vivo imaging of transplanted AF750-labeled donor cells in various allogeneic BMT models at 24 h after BMT.

We attempted to visualize the biodistribution of allogeneic BMT models with C57BL/6 (as donors) and Balb/c nu/nu mice (as recipients) to analyze the onset of aGVHD. Because the depletion of donor T cells abrogates GVHD [15], TCD-BMT (nonGVHD) and TCD-BMT plus Sp-MNCs (GVHD) transplantation models were established. In allogeneic BMT and the GVHD models, the fluorescent signals from the reticuloendothelial system were very high at 24 h after BMT (Fig. 6A, right top and bottom panels). The tissues with a high fluorescent signal were confirmed by ex vivo imaging 24 h after BMT (Fig. 6B). Among the reticuloendothelial system, the fluorescence was lowest in the nonGVHD model and highest in the GVHD model (Fig. 6B). These results indicate that the presence of a T-cell population in the donor cells causes homing to the reticuloendothelial system and that the TCD donor cells predominantly homed to BM. The fluorescent signal was very weak in the intestinal tract in all allogeneic BMT models (Fig. 6B, bottom row).

## Discussion

To our knowledge, this study is the first report describing the use of in vivo real-time imaging to visualize the distribution dynamics of transplanted BM cells in the entire body during the initial phase after BMT using the NIRF method. Achieving in vivo imaging at a very early stage after BMT was previously difficult because the small number of cells that escape the reticuloendothelial system result in minute cell distributions within each organ. In this study, we labeled the surface of transplanted cells with NIRF and successfully demonstrated the altered distribution dynamics of transplanted cells in the initial phase. We could noninvasively track transplanted cells circulating in the entire body during the initial stage after BMT because of the following reasons: (1) NIRF illumination with higher light propagation and lower attenuation through the tissue was used as both excitation and emission light for imaging [16]; (2) homogeneous labeling on the cell surface with NIRF dyes was achieved despite a variety of cell populations; (3) NIRF-labeled transplanted donor cells had a constant MFI, detectable in vivo over a 24-h period; (4) Balb/c nu/nu mice (hairless mice) were used to enhance NIRF signal detection sensitivity; and (5) our original fasting protocol successfully excluded autofluorescence derived from food particles, the major obstacle in precise imaging of the abdomen.

Balb/c nu/nu mice lack the *whn* gene [17], [18], which encodes a forkhead/winged-helix transcription factor confined to expression in the thymic epithelium, epidermis, and hair follicles. As a result of the *whn* gene deficiency, Balb/c nu/nu mice lack the thymus and post-thymic mature T cells [19]. However, this gene is not expressed in HSCs [20], and reduction in the function of antigen-presenting cells such as macrophages and dendritic cells of Balb/c nu/nu mice has not been reported. Therefore, there is little possibility that their genetic defect influences early transplantation events such as homing of HSCs or antigen presentation in GVHD.

Abdominal autofluorescence caused by food disturbs precise evaluation of in vivo imaging. To suppress autofluorescence from food particles in the GI tract, all mice were fasted according to our original fasting protocol prior to in vivo imaging (Supplemental Fig. S1). As a result of fasting, the autofluorescence of the GI tract was dramatically attenuated in vivo and ex vivo (Supplemental Fig. S6). Obvious fasting-related organ damage was not apparent in these mice (Supplemental Fig. S6).

Homing of AF750-labeled donor cells in the tibiae was detected less than 1 h after BMT in the congenic BMT model. Signals from the tibia were noted at 3 h after transplantation and continued to rise until 24 h after transplantation. IBM-BMT is a method of transplanting HSCs and stromal cells directly into niches in the BM cavity [21], [22], and the kinetics and homing of the transplanted cells to all the bones of the body after IBM-BMT have not been determined previously. In this study, we elucidated that most of the transplanted cells remained within the injected tibia, while some cells entered the peripheral circulation though the BM sinus of the tibia, and cells trapped in the lung were observed 5 min after IBM-BMT. Moreover, circulating cells started migrating to the backbone and the collateral tibia of the injected site 1 h after IBM-BMT.

FACS analysis using ICR/EGFP as donors revealed that transplanted donor cells started cell division more than 24 h after BMT and that hematopoietic reconstitution in Balb/c nu/nu mice recipients began approximately 72 h after BMT (Fig. 5), indicating that donor cell homing must have had started within 24 h after BMT. Therefore, it is very important to understand both the homing process of HSCs and mechanisms of engraftment failure for accurate assessment of transplanted donor cell distribution during the very initial phase after BMT.

With regard to aGVHD, Peyer's patches (gut-associated secondary lymphoid organs) are the initial sites of aGVHD [23], [24], and it has been previously reported that the infiltration of donor cells into Peyer's patches rapidly increased around 3 days after BMT in murine GVHD models [8]. Furthermore, splenectomy and prevention of T-cell entry into all secondary lymphoid organs with αMAdCAM-1 and αCD62L monoclonal antibodies abrogated the onset of gut GVHD [25]. Considering these reports, gut GVHD would begin within 72 h after BMT, and involvement of all secondary lymphoid organs may be necessary to evoke gut GVHD. Our results regarding the allogeneic BMT and GVHD models revealed that the donor cells were barely detected in the intestinal tract within 24 h, and very few instances of direct homing to Peyer's patches were detected in the allogeneic BMT models. On the other hand, a large number of cells were found trapped in the lung, liver, spleen, and superficial lymph nodes within 24 h after BMT (Fig. 6). Considering these observations, it seems that a larger number of cells was present in the reticuloendothelial system than in Peyer's patches until 24 h after BMT and that the secondary infiltration of T cells from secondary lymphoid organs to Peyer's patches is also considered to be very important for induction of gut GVHD.

The mechanisms of hematopoietic cell homing have not been revealed. It is assumed that the homing of hematopoietic cells is caused by molecular interactions of transplanted cells with their niche components [26], [27] and that homing processes are varied among hematopoietic cell populations [28]. As for lymphocytes, high endothelial venules in lymph nodes or secondary lymphoid tissues contribute to continuous immune surveillance by supporting lymphocyte recruitment from the blood [29], [30]. Our results clearly revealed that in the initial phase after BMT, the cell distributions were varied among BMT models. For example, in GVHD models, a T-cell population dominantly homed to the reticuloendothelial system, whereas TCD donor cells dominantly homed to BM (Fig. 6B). Using TCD BM-MNCs, which contained less lymphocytes and more hematopoietic cells including HSCs, we demonstrated that transplanted cells primarily homed to BM and secondarily homed to lymphoid organs, including the reticuloendothelial system (Fig. 6B).

In this study, we labeled the surface of transplanted cells with NIRF and successfully demonstrated their altered distribution dynamics during the initial phase. There are several transgenic murine models with unique HSCs or BM microenvironments. Our method may be useful to obtain spatiotemporal information of the donor cell homing process in these mice. Together with other optical imaging techniques such as bioluminescence imaging, we believe that this method would provide a means to study the pivotal process of complications associated with BMT, such as engraftment failure and GVHD, and would contribute to improvement of therapeutic outcomes of BMT.

## Supporting Information

Figure S1 (A) Our original fasting protocol. (B) Autofluorescence of the feed and stool of Balb/c mice. Autofluorescence of each solution (C) and compositions (D) of solutions. (E) Evaluation of % body weight change of fasting Balb/c mice (OS-1 versus H2O) (n = 5). (F) Survival curve of mice in (E) (n = 5).
(2.29 MB TIF)

Figure S2 The proportions of sub-G1 fractions of Balb/c nu/nu BM-MNCs was analyzed by FACS at 2 h after labeling (n = 5). DNA content was analyzed as previously described [13]. The population of cells with degraded genomic DNA (sub-G1 fraction) was identified. Values are mean rates ± SEM for the indicated concentrations.
(1.72 MB TIF)

Figure S3 Viable Balb/c Sp-MNCs were analyzed by a modified MTT assay. Various concentrations of Cy5.5- or AF750-labeled Sp-MNCs (1×105) were seeded in a 96-well plate with phenol red-free RPMI 1640 medium containing CD3/28 beads and mIL2 (n = 5). The cells were then incubated for the indicated times. At the end of the culture period, the viable cell number was analyzed as previously described [12] with slight modifications. The absorbance (450 nm) was measured 3 h after adding the cell-counting reagent. The experiments were done in at least triplicate, and results are presented as the mean relative OD ± SEM.
(1.73 MB TIF)

Figure S4 The proportions of sub-G1 fractions caused by a single fraction of near-infrared excitation and emission. NIRF-labeled or unlabeled Balb/c nu/nu BM-MNCs were excited for 5 s using the IVIS (Cy5.5: 640 nm, AF750: 710 nm) and the sub-G1 fractions were analyzed by FACS at 2 h after excitation (n = 5).
(1.77 MB TIF)

Figure S5 FACS analysis for the decrement of mean fluorescence intensity (MFI) by repeated excitations. NIRF-labeled Balb/c nu/nu BM-MNCs were excited for 5 s per excitation using the IVIS (n = 5). The MFIs of NIRF-labeled BM-MNCs were analyzed by FACS at 2 h after repeated excitation for the indicated times.
(0.89 MB TIF)

Figure S6 (A,B) Autofluorescence in the abdomen was significantly reduced by the original fasting protocol. (C) Histological analyses (H&E staining) of the indicated organs in fasted mice on day 1. Fasting-related organ damage was not apparent in the fasting mice. Bar  = 100 µm.
(2.62 MB TIF)

Figure S7 In vivo imaging of transplanted Cy5.5-labeled donor cells. In vivo imaging of Cy5.5-labeled Balb/c nu/nu BM-MNCs with mice in the supine and prone positions. Signals for the transplanted Cy5.5-labeled cells were seen in the backbone and the tibia. The areas indicated by squares are magnified and are shown below each image.
(3.57 MB TIF)

Figure S8 In vivo imaging of transplanted AF750-labeled donor cells. Conditioned recipients were injected with indicated solutions.
(2.23 MB TIF)

Figure S9 Donor EGFP+ cells appeared in the peripheral blood at 72 h with similar fluorescence intensity to that in the BM. Purple, donor cells; Black, recipient cells.
(1.15 MB TIF)
