# Supplementary figures and images for: Noninvasive Tracking of Donor Cell Homing by Near-Infrared Fluorescence Imaging Shortly after Bone Marrow Transplantation
Source: PLoS One. 2010 Jun 14;5(6):e11114. doi: 10.1371/journal.pone.0011114 (PMC2885427; doi:10.1371/journal.pone.0011114)

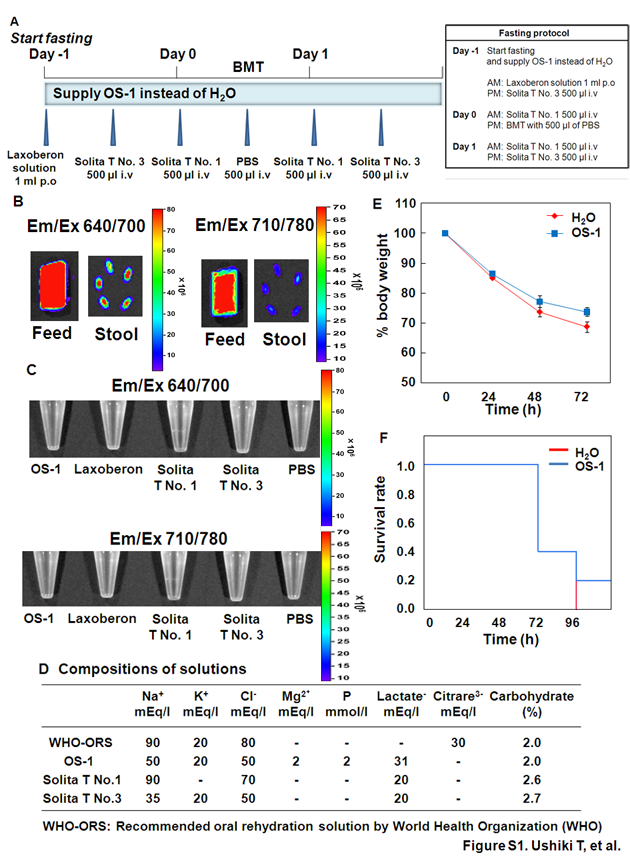

Supplement: Figure S1 — (A) Our original fasting protocol. (B) Autofluorescence of the feed and stool of Balb/c mice. Autofluorescence of each solution (C) and compositions (D) of solutions. (E) Evaluation of % body weight change of fasting Balb/c mice (OS-1 versus H2O) (n = 5). (F) Survival curve of mice in (E) (n = 5). (2.29 MB TIF) [file pone.0011114.s001.tif]

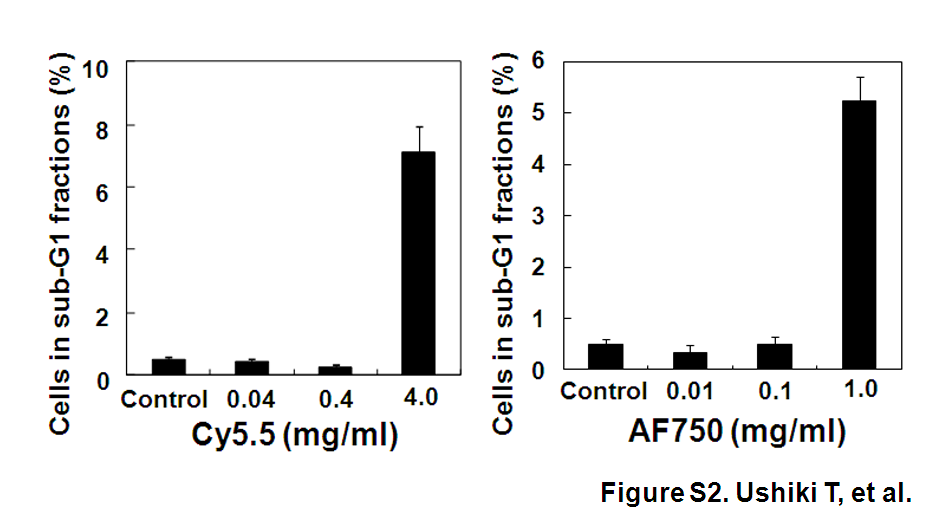

Supplement: Figure S2 — The proportions of sub-G1 fractions of Balb/c nu/nu BM-MNCs was analyzed by FACS at 2 h after labeling (n = 5). DNA content was analyzed as previously described [13]. The population of cells with degraded genomic DNA (sub-G1 fraction) was identified. Values are mean rates ± SEM for the indicated concentrations. (1.72 MB TIF) [file pone.0011114.s002.tif]

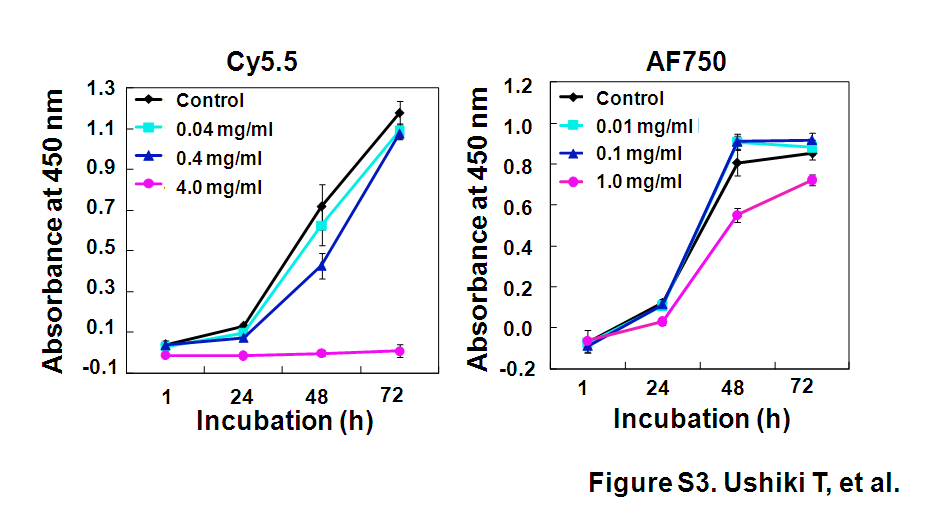

Supplement: Figure S3 — Viable Balb/c Sp-MNCs were analyzed by a modified MTT assay. Various concentrations of Cy5.5- or AF750-labeled Sp-MNCs (1×10^5^) were seeded in a 96-well plate with phenol red-free RPMI 1640 medium containing CD3/28 beads and mIL2 (n = 5). The cells were then incubated for the indicated times. At the end of the culture period, the viable cell number was analyzed as previously described [12] with slight modifications. The absorbance (450 nm) was measured 3 h after adding the cell-counting reagent. The experiments were done in at least triplicate, and results are presented as the mean relative OD ± SEM. (1.73 MB TIF) [file pone.0011114.s003.tif]

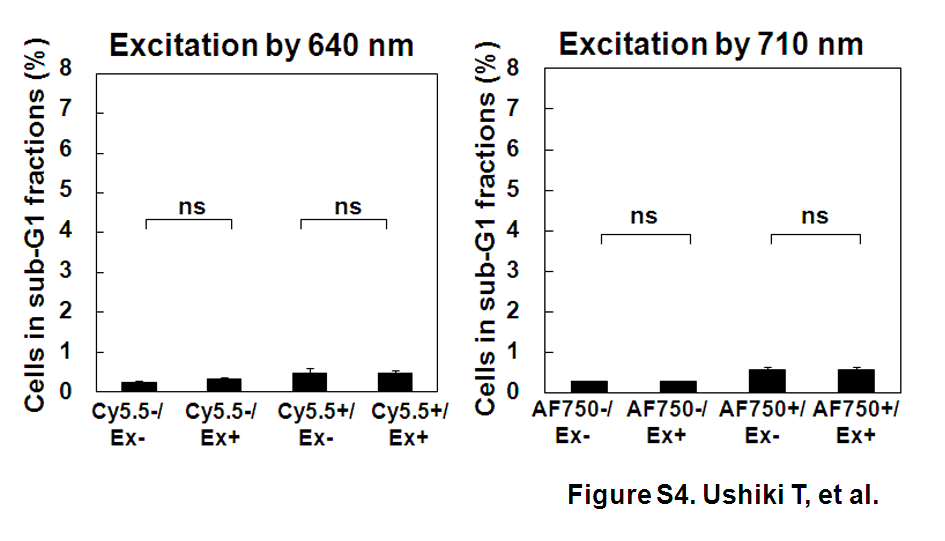

Supplement: Figure S4 — The proportions of sub-G1 fractions caused by a single fraction of near-infrared excitation and emission. NIRF-labeled or unlabeled Balb/c nu/nu BM-MNCs were excited for 5 s using the IVIS (Cy5.5: 640 nm, AF750: 710 nm) and the sub-G1 fractions were analyzed by FACS at 2 h after excitation (n = 5). (1.77 MB TIF) [file pone.0011114.s004.tif]

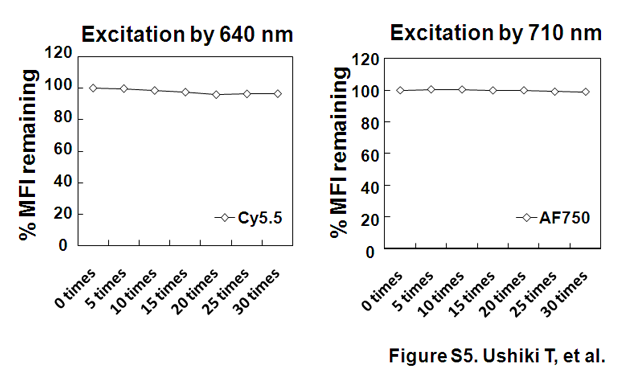

Supplement: Figure S5 — FACS analysis for the decrement of mean fluorescence intensity (MFI) by repeated excitations. NIRF-labeled Balb/c nu/nu BM-MNCs were excited for 5 s per excitation using the IVIS (n = 5). The MFIs of NIRF-labeled BM-MNCs were analyzed by FACS at 2 h after repeated excitation for the indicated times. (0.89 MB TIF) [file pone.0011114.s005.tif]

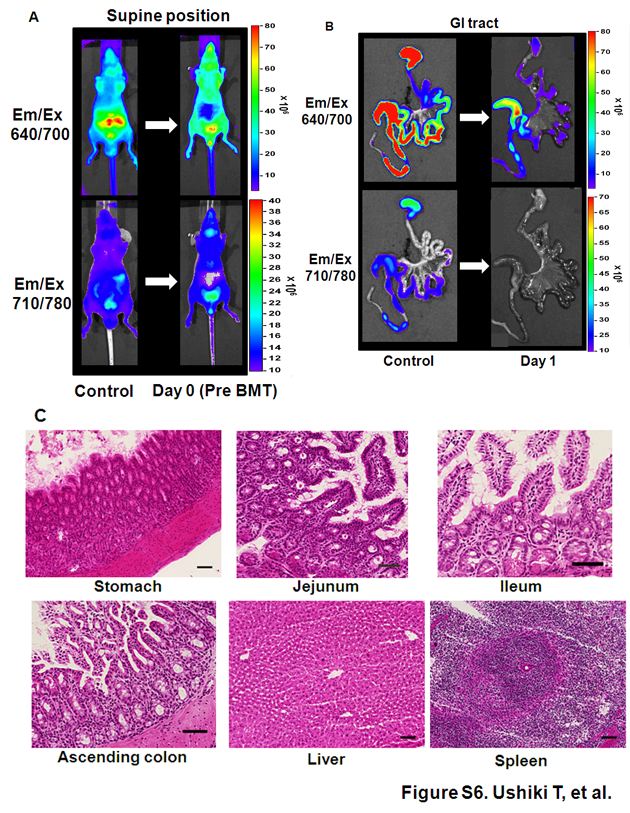

Supplement: Figure S6 — (A,B) Autofluorescence in the abdomen was significantly reduced by the original fasting protocol. (C) Histological analyses (H&E staining) of the indicated organs in fasted mice on day 1. Fasting-related organ damage was not apparent in the fasting mice. Bar = 100 µm. (2.62 MB TIF) [file pone.0011114.s006.tif]

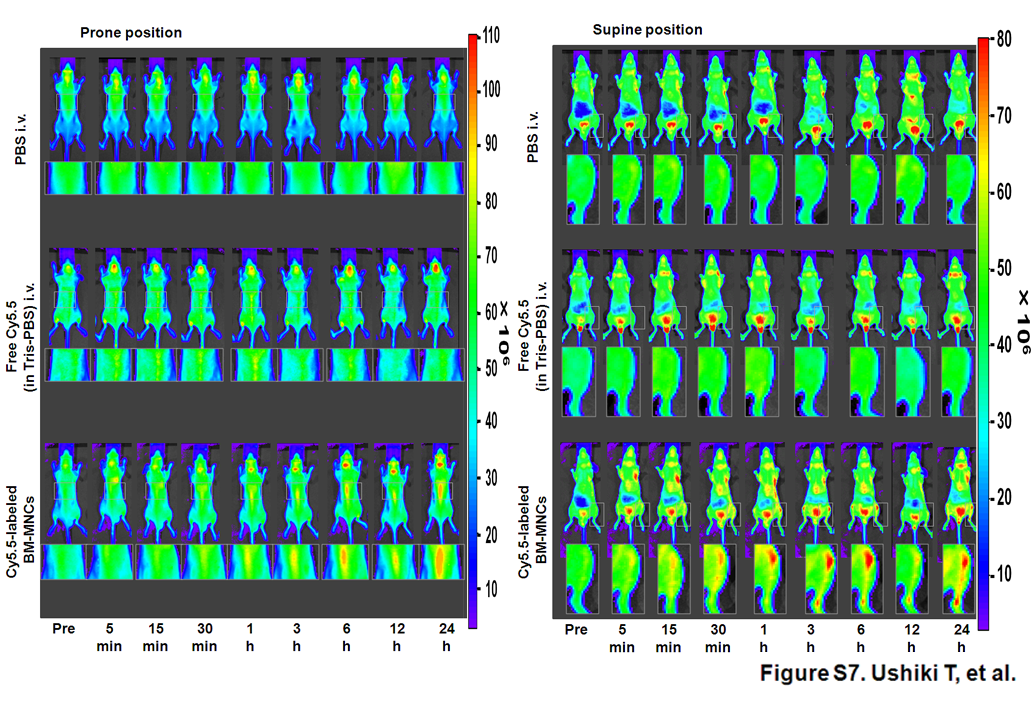

Supplement: Figure S7 — In vivo imaging of transplanted Cy5.5-labeled donor cells. In vivo imaging of Cy5.5-labeled Balb/c nu/nu BM-MNCs with mice in the supine and prone positions. Signals for the transplanted Cy5.5-labeled cells were seen in the backbone and the tibia. The areas indicated by squares are magnified and are shown below each image. (3.57 MB TIF) [file pone.0011114.s007.tif]

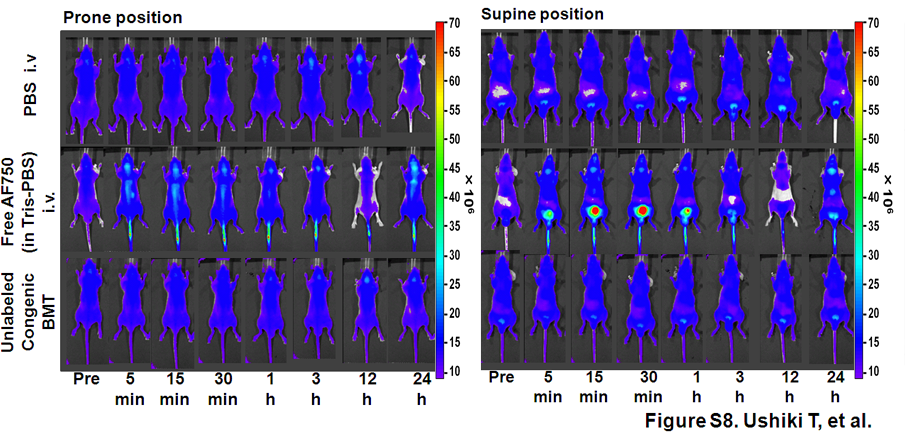

Supplement: Figure S8 — In vivo imaging of transplanted AF750-labeled donor cells. Conditioned recipients were injected with indicated solutions. (2.23 MB TIF) [file pone.0011114.s008.tif]

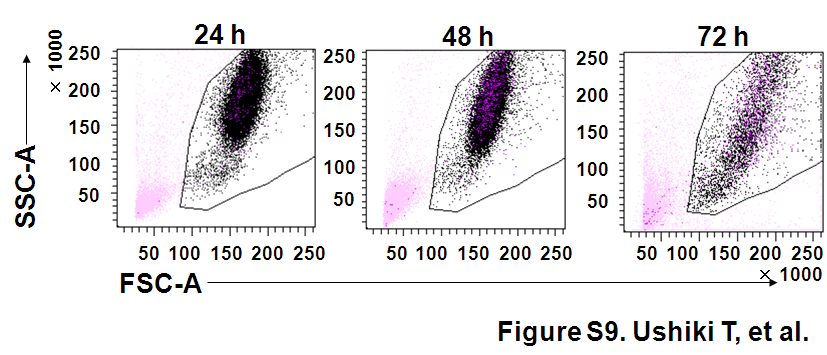

Supplement: Figure S9 — Donor EGFP+ cells appeared in the peripheral blood at 72 h with similar fluorescence intensity to that in the BM. Purple, donor cells; Black, recipient cells. (1.15 MB TIF) [file pone.0011114.s009.tif]
